# Plasma lipidomic signatures reveal age-associated patterns of septic shock risk and immune dysregulation in sepsis

**DOI:** 10.3389/fimmu.2025.1659425

**Published:** 2025-11-19

**Authors:** Yuhan Sun, Ke Lin, Ling Wang, Jingwen Ai, Jingjing Zhao, Ruiwen Sun, Xiaoyang Cheng, Yanmin Wan, Peng Cui, Sen Wang, Jing Wu, Jialin Jin

**Affiliations:** 1 Department of Infectious Diseases, Shanghai Key Laboratory of Infectious Diseases and Biosafety Emergency Response, National Medical Center for Infectious Diseases, Huashan Hospital, Fudan University, Shanghai, China; 2 Shanghai Omicsolution Co., Ltd., Shanghai, China; 3 Shanghai Sci-Tech Inno Center for Infection & Immunity, Shanghai, China

**Keywords:** sepsis, lipidomics, aging, immunity, shock

## Abstract

**Background:**

Sepsis is associated with significant lipidomic disturbances, but age-associated lipidomic patterns remain poorly characterized. Given the links between aging, immune dysfunction, and metabolic dysregulation, defining age-specific lipid profiles could improve sepsis risk assessment. This study investigates age-stratified lipidomic signatures in sepsis and identifies biomarkers for clinical severity.

**​Methods:**

We prospectively enrolled 62 sepsis patients (21 <65 years, 41 ≥65 years) and 40 healthy controls. Plasma lipidomics was performed via untargeted LC-MS/MS, identifying 1,277 lipid species across 38 subclasses. Principal component analysis (PCA) and consensus clustering were used to assess lipidomic differences and define patient subtypes. Correlations between lipid subclasses, clinical severity (SOFA scores), and immune cell subsets were analyzed. An age-adjusted risk stratification model was developed to assess septic shock and mortality risk (AUC analysis).

**​Results:**

Sepsis patients exhibited reduced phosphatidylcholine (PC), cholesteryl ester (CE), and lysophosphatidylcholine (LPC) levels (all P<0.05). Clustering revealed four lipidomic patterns, with Cluster 4 distinguishing two sepsis subtypes (C1, C2). Subtype C2 had higher septic shock incidence (57.1% vs. 14.8%, P = 0.0013) and downregulation of 92 lipids, 35 of which strongly correlated with SOFA scores. A risk stratification model incorporating six key lipids (LPC(19:0), PC(P-19:0), SM 32:3;2O(FA 16:3), PC(P-20:0), PC(O-18:1/20:3), CE(15:0)) and age accurately predicted septic shock (AUC: 0.87 training, 0.82 validation) and mortality risk in elderly patients. PC levels correlated with monocytes, while CE and LPC associated with complement proteins and CD8+ T cells.

**​Conclusions:**

Our lipid-based model effectively predicts septic shock and mortality, particularly in elderly sepsis patients. Age-associated lipid alterations (PC, LPC, CE reduction) correlate with disease severity and immune dysregulation, suggesting distinct lipid-immune mechanisms in younger vs. elderly patients. These findings support lipidomics as a tool for sepsis risk stratification and personalized therapy.

## Introduction

Sepsis is a complex, life-threatening condition characterized by a dysregulated host response to infection, leading to systemic inflammation and potential multiorgan failure (1–3). It has emerged as a significant global health challenge, with high incidence and mortality rates, particularly among older adults (2, 4). Plasma lipidomics, which involves comprehensive analysis of lipid molecules in biological samples, holds great potential for elucidating the metabolic and immune regulatory mechanisms underlying the onset and progression of sepsis (5, 6), thereby reflecting the host’s metabolic status and immune function (7, 8). However, current research on the lipidomic profiles of sepsis patients remains insufficient, particularly due to the lack of systematic age-stratified analyses, which hinders the identification of age-specific metabolic and immune alterations in these patients (9, 10).

Previous studies have focused primarily on targeted measurements of a select few lipid species (11, 12), failing to capture the broader lipidomic landscape. Additionally, while the literature indicates metabolic disturbances in critically ill patients (13, 14), there is a lack of age-stratified investigations into the overall relationship of the plasma lipidome with disease severity and immune cell dynamics (15). Elderly patients, due to physiological decline, immunosenescence, and metabolic changes, typically exhibit heightened susceptibility to infections (16). The age-associated deterioration of immune function further complicates disease management and leads to significantly worse clinical outcomes (17). Therefore, conducting age-stratified investigations into the lipid metabolic characteristics of sepsis patients is of paramount importance for elucidating disease mechanisms and optimizing clinical management strategies (18).

The interplay between lipid metabolism and the immune response is critical to the progression and outcomes of sepsis (19). We hypothesized that there are significant age-specific lipid profile changes in patients with sepsis, and that these changes correlate with disease severity and immune cell function. Lipids serve not only as fundamental structural components of cellular membranes but also as critical modulators of inflammatory and immune signaling pathways (20, 21). Age-specific alterations in lipid metabolic profiles may be intrinsically linked to divergent immune regulatory mechanisms across age groups. Elucidating these changes could yield clinically actionable biomarkers for improved diagnosis and prognostic prediction, thereby advancing the development of personalized therapeutic strategies (22). To validate this hypothesis, we performed comprehensive untargeted liquid chromatography-tandem mass spectrometry (LC-MS/MS)-based plasma lipidomic profiling of both sepsis patients and healthy controls, with particular focus on identifying age-stratified lipidomic variations and their clinical correlations.

## Methods

### Study population and group allocation

This study was conducted in the Department of Infectious Diseases at Huashan Hospital, Fudan University, from September 2022 to September 2023. The research protocol was approved by the Ethics Review Committee of Huashan Hospital. Inpatients with suspected infections were enrolled, and samples were collected on the day of fever onset. Patients meeting the Sepsis-3 criteria were included in the sepsis group. Patients aged 65 years or older were classified into the elderly sepsis group (asepsis), whereas those younger than 65 years were classified into the younger sepsis group (ysepsis). Additionally, we enrolled 20 elderly healthy controls (aHC) and 20 young healthy controls (yHC) from the health examination center.

Among all sepsis patients, those who exhibited persistent hypotension despite adequate fluid resuscitation and required vasopressor support to maintain a mean arterial pressure (MAP) of ≥ 65 mmHg, along with serum lactate levels > 2 mmol/L, were classified into the septic shock group. Healthy controls were sampled from community health screenings. Informed consent was obtained from all participants.

All blood samples were collected at hospital admission, prior to the administration of any antibiotic therapy. Demographic, clinical, microbiological, and laboratory data were collected by dedicated research physicians. Physiological measurements, vital signs, and disease severity scores, including the Sequential Organ Failure Assessment (SOFA) score, were recorded.

### Sample preparation

The samples were thawed at 4 °C, and 100 µL of serum was transferred to a new tube. To this mixture, 200 µL of methanol (MeOH) and 200 µL of acetonitrile (ACN) were added. The mixture was vortexed for 30 seconds and then sonicated in an ice-water bath for 10 minutes to ensure thorough mixing. To precipitate the proteins, the samples were incubated for 1 hour at -20°C. Following incubation, the samples were centrifuged at 13,000 rpm for 15 minutes at 4°C, and the supernatant was carefully collected. The supernatant was placed in a rotary evaporator at 4°C and evaporated to dryness. The dry extracts were then resuspended in 100 µL of a 1:1 mixture of acetonitrile and water. Vortexed for 30 seconds and then sonicated in an ice-water bath for an additional 10 minutes. The resulting mixture was centrifuged at 13,000 rpm for 15 minutes at 4°C, and the supernatant was collected and stored at -80°C until further analysis.

### LC–MS/MS analysis

LC-MS/MS analysis was performed via a high-performance liquid chromatography (HPLC) system (1290 Series, Agilent Technologies, USA) coupled to a Bruker timsTOF mass spectrometer equipped with an electrospray ionization (ESI) source (Bruker Daltonik, Germany). A Phenomenex Kinetex C18 column (particle size: 1.7 µm; dimensions: 100 mm length × 2.1 mm inner diameter) was utilized for liquid chromatography separation, with the column temperature maintained at 55°C. Mobile phase A consisted of water/acetonitrile (6:4, v/v) with 10 mM ammonium formate, while mobile phase B consisted of isopropanol/acetonitrile (9:1, v/v) with 10 mM ammonium formate. Flow rate: 0.30 mL/min. Injection volume: 2 µL. Run time: 18 minutes.

The elution gradient was programmed as follows: 40% B from 0 to 1.5 minutes, a linear increase from 40% B to 85% B from 1.5 to 10.5 minutes, holding at 85% B from 10.5 to 14.0 minutes, transitioning from 85% B to 100% B from 14.0 to 14.1 minutes, holding at 100% B from 14.1 to 15.0 minutes, decreasing back to 40% B within 0.2 minutes, and maintaining at 40% B for 2.8 minutes for column equilibration. The flow rate was set at 0.3 mL/min, and a 2 µL injection volume was used for biological samples in positive mode.

For mass spectrometry acquisition, the PASEF-DDA scan mode was employed to detect ions with m/z values ranging from 100 Da to 1350 Da and mobility values ranging from 0.55 to 1.90 V·s/cm². The MS parameters were as follows: capillary voltage was set to +4500 V; nebulizer pressure was 2.2 bar; dry gas flow was 10.0 L/min at a temperature of 220 °C; the number of PASEF MS/MS scans was set to 2; the ramping time was 100 ms; the total cycle time was 0.32 s; the charge range was 0–1; the absolute threshold was 100 counts; active exclusion was enabled, with previously targeted ions released after 0.1 min; the isolation window was set to 1.2 Da; and the collision energy was set to 40 eV.

### Lipidomic profiling

Lipid molecular annotation and raw data preprocessing of the mass spectrometry data were performed using the online tool Lipid4DAnalyzer (http://lipid4danalyzer.zhulab.cn/). The final lipid metabolite list and data matrix were obtained. To ensure the reliability of subsequent statistical analyses, the raw data were preprocessed as follows before formal analysis: Feature peaks with a non-zero value occurrence rate of >50% across all samples were retained. Missing values in the remaining feature peaks were imputed using the K-Nearest Neighbors (KNN) method. Normalization was performed using quality control (QC) samples, and feature peaks with a relative standard deviation (RSD) >30% in QC samples were excluded. Through this workflow, a total of 1277 lipid molecules were annotated. Based on the LIPID MAPS (Lipid Metabolites and Pathways Strategy) classification system, these 1277 lipid molecules were categorized into 38 lipid subclasses according to their structures.

### Statistical analysis

All statistical analyses were performed using R statistical software (version 4.3.2), with data visualization primarily implemented via the ggplot2 package (version 3.5.1).

We conducted PCA using the FactoMineR package (version 2.11) to reduce dimensionality. The Wilcoxon rank-sum test was applied to assess the significance of inter-group distribution differences along PCA principal coordinates, thereby elucidating global lipidomic profile disparities.

Individual lipid species were compared between groups using two-sample t-tests, with statistical significance defined as P < 0.05. To address multiple testing, all P-values were adjusted via the Benjamini-Hochberg false discovery rate (FDR) method.

Linear regression models (ordinary least squares, OLS) were constructed to evaluate relationships between lipid levels and SOFA scores at two levels:

Lipid subclass level: Expression values of all lipids within a subclass were summed, followed by log_2_-transformation and z-score normalization to mitigate distributional bias.

Individual lipid level: Raw expression values were directly log_2_-transformed and z-score normalized.

Normalized lipid subclass/individual lipid values served as independent variables, with SOFA scores as the dependent variable. Regression coefficients and their significance (P-values) were computed.

​Lasso regression​ was employed to identify SOFA-associated key lipids.A ​logistic regression model​ integrating selected lipids and patient age was developed.Model performance was evaluated via ​5-fold cross-validation​ (200 repeats) and quantified using receiver operating characteristic (ROC) curves and area under the curve (AUC).

## Results

### Study population

**Figure 1:**
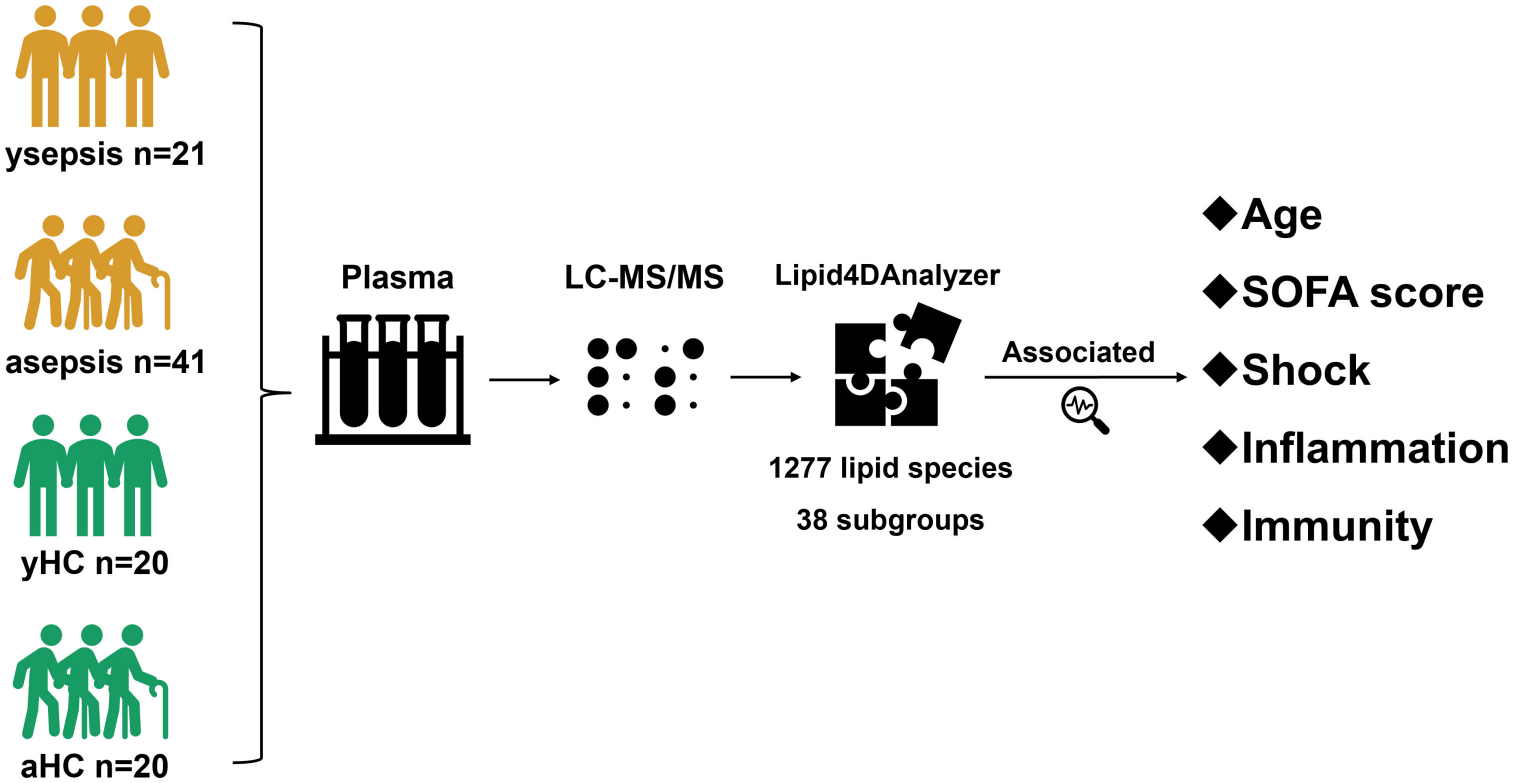
Research process.

We enrolled 62 hospitalized patients diagnosed with sepsis and 40 healthy individuals as controls. Among the sepsis patients, 41 were older adults (≥65 years), and 21 were younger adults (<65 years). The control group included 20 older adults and 20 younger adults. (**Figure 1**) The mean age of young sepsis was 40 (28-46), with a male prevalence of 48%. The average age of older sepsis cases was 77 (69-82), of which 63% were male. 44% of older sepsis cases had hypertension and 49% had diabetes. In young sepsis, 19% had hypertension and 33% had diabetes. In the sepsis cohort, the most common pathogen was *Klebsiella pneumoniae* (n=25), followed by *Acinetobacter baumannii* (n=14), *Staphylococcus aureus* (n=8), and Enterococcus spp. (n=7). Seventeen patients had mixed infections. The incidence of shock in sepsis patients was 38.7% (24/62), and 21 patients had in-hospital mortality (**Table 1**).Patients with malignancies or autoimmune diseases were excluded.

**Table 1:** Study population characteristics.

Patient characteristics	The young-adult cohort (<65y)		The elder cohort(≥65y)	
	ysepsis	yHC	asepsis	aHC
N	21	20	41	20
Gender, male (%)	10 (48)	4 (20)	26 (63)	10 (50)
Age, median(IQR)	40 (28-46)	44 (32-57)	77 (69-82)	75 (70-79)
Comorbidities				
Obesity BMI>28,N(%)	2 (10)	0 (0)	0 (0)	0 (0)
Hypertension,N(%)	4 (19)	0 (0)	18 (44)	0 (0)
Diabetes,N(%)	7 (33)	0 (0)	20 (49)	0 (0)
Site of infection				
Lung,N(%)	12 (57)	0 (0)	35 (85)	0 (0)
Pathogenic bacteria				
K. pneumoniae,N(%)	5 (24)	0 (0)	12 (29)	0 (0)
A. baumannii,N(%)	0 (0)	0 (0)	6 (15)	0 (0)
S. aureus,N(%)	2 (10)	0 (0)	3 (7)	0 (0)
Enterococcus,N(%)	3 (14)	0 (0)	1 (2)	0 (0)
Multipathogen,N(%)	5 (24)	0 (0)	12 (29)	0 (0)
Shock,N(%)	8 (36)	0 (0)	16 (40)	0 (0)
Death,N(%)	4 (19)	0 (0)	17 (41)	0 (0)

The higher proportion of males in the elderly sepsis group (63% vs. 48% in young sepsis) may reflect both the demographic characteristics of severe sepsis in aging populations and the potentially higher susceptibility of aged males to severe infections.

### Lipid landscape differences between aHC and yHC

**Figure 2:**
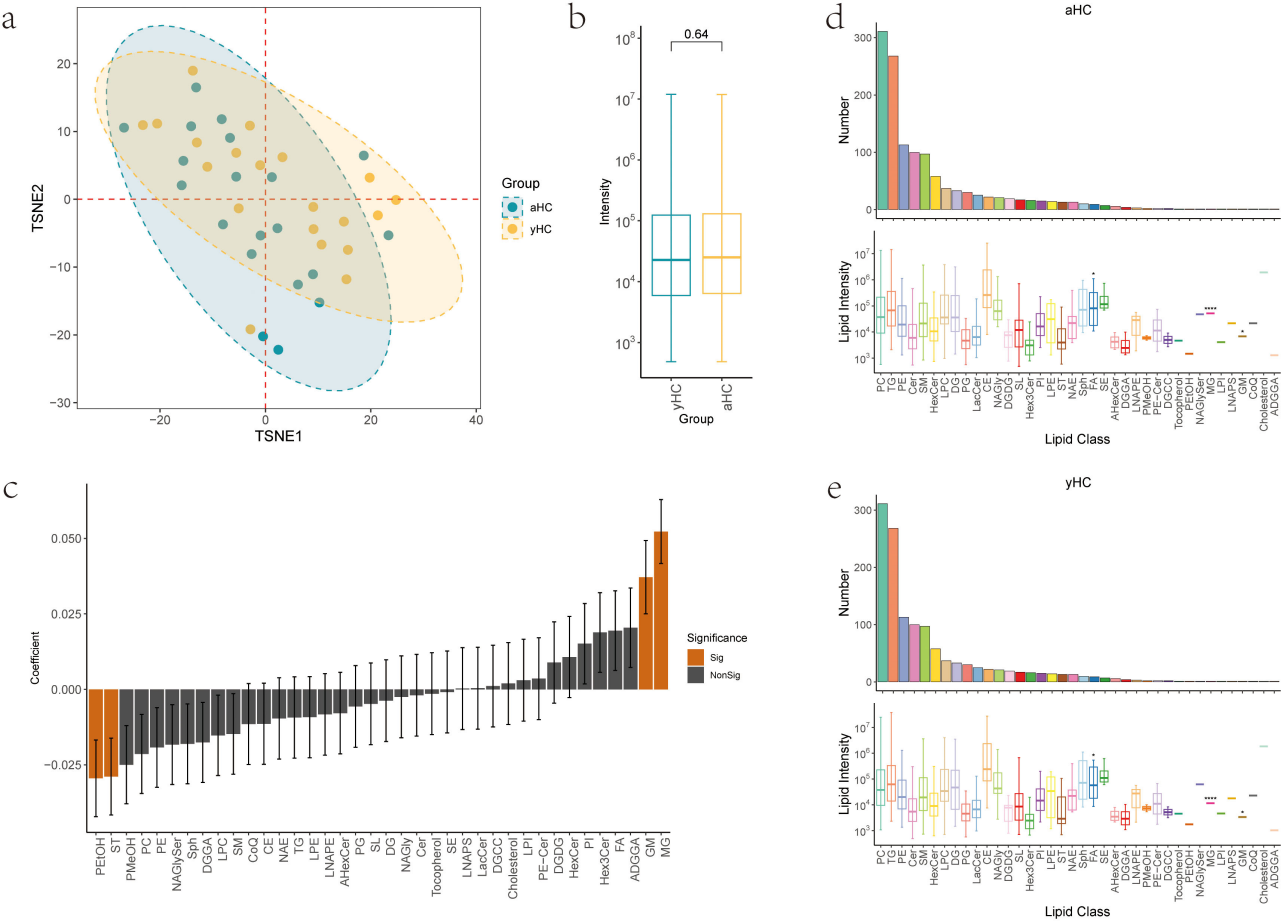
Lipid landscape differences between aHC and yHC. **(a)** t-SNE visualization of lipid profiles, combining young (yHC, yellow) and aged (aHC, blue) healthy cohorts. Each dot represents a sample. TSNE1: P = 0.66. **(b)** Boxplots comparing global lipid abundance between yHC and aHC, showing no significant difference (P = 0.64). **(c)** Linear correlations between lipid subclasses and age. PEtOH and ST show significant negative correlations (PEtOH: coeff=-0.029, P = 0.026; ST: coeff=-0.028, P = 0.028), while MG and GM show significant positive correlations (MG: coeff=0.052, P<0.001; GM: coeff=0.037, P = 0.004). Orange indicates P<0.05. **(d, e)** Lipid subclass distribution in aHC and yHC. Bar plots: molecular counts per subclass. Boxplots: abundance distribution per subclass. FA, MG, and GM are more abundant in aHC (P = 0.018, P<0.001, P = 0.045). *P<0.05, ****P<0.0001. aHC, aged healthy; yHC, young healthy.

We first examined the lipidome of healthy baseline samples, defined as samples from individuals without any self-reported acute disease, although potentially including asymptomatic chronic conditions such as prediabetes or undiagnosed diseases. We analyzed 40 healthy baseline samples and conducted t-distributed stochastic neighbor embedding (t-SNE) analysis to characterize lipid abundance differences between younger and older healthy adults. The t-SNE visualization showed substantial overlap in lipidomic profiles between aHC and yHC groups (t-SNE1 P = 0.66), suggesting relative stability of the plasma lipidome in healthy aging (**Figure 2a**). Box plot analysis revealed no significant difference in overall lipid abundance between the older (aHC) and younger (yHC) healthy groups (P = 0.64) (**Figure 2b**), indicating similar lipid distributions across ages under healthy conditions.

To facilitate interpretation, we classified the 1,277 identified lipid molecules into 38 subcategories on the basis of structure, including PEtOH, ST, ADGGA, AHexCer, CE, Cer, cholesterol, CoQ, DG, DGCC, DGDG, DGGA, FA, GM, Hex3Cer, HexCer, LacCer, LNAPE, LNAPS, LPC, LPE, LPI, MG, NAE, NAGly, NAGlySer, PC, PE, PE-Cer, PG, PI, PMeOH, SE, SL, SM, Sph, TG, and Tocopherol. Assuming that lipid levels might vary by age, we applied a linear mixed model and found a significant negative correlation with age for most lipid categories, particularly PEtOH (coefficients=-0.029, P = 0.026) and ST (coefficients=-0.028, P = 0.028). MG and GM levels were significantly positively correlated with age (MG, coefficient=0.052, P<0.001; GM, coefficient=0.037, P = 0.004) (**Figure 2c**). The 38 lipid subcategories ranked similarly in terms of abundance between the aHC and yHC, with no major differences in lipid types between the age groups (**Figures 2d, e**). FA, MG, and GM levels were higher in older healthy adults than in younger adults (P = 0.018, P<0.001, P = 0.045). PC was the most abundant subcategory, comprising 311 lipid species, whereas CE, Sph, and FA had broader dynamic abundance ranges.

### Sepsis-associated lipid signatures

**Figure 3:**
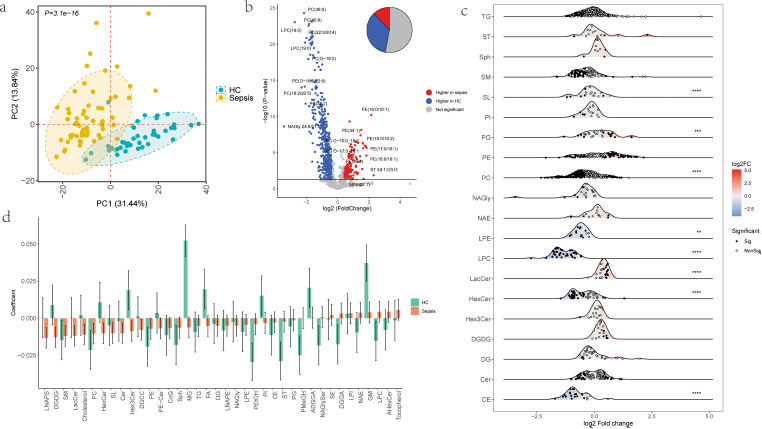
Lipid landscape differences between HC and sepsis. **(a)** PCA plot of HC and sepsis groups. Each point represents a sample, colored by group. Axes show PC1 and PC2. P = 3.1e−16. **(b)** Volcano plot of lipid abundance differences. Points represent lipids, colored by significance. X-axis: fold change; Y-axis: -log10 P-value. Red: upregulated in sepsis; blue: downregulated. **(c)** Correlation of lipid subclasses with age. Green: HC; orange: sepsis. Y-axis: correlation coefficient. **(d)** Ridge plot of lipid subclasses with ≥10 molecules. X-axis: log2 fold change; * indicates significant differences (|FC|>1.3, P<0.05). Points represent individual lipids within subclasses (Y-axis), colored by significance (white: significant; black: nonsignificant). *P<0.05, **P<0.01, ***P<0.001, ****P<0.0001.

We used principal component analysis (PCA) to explore plasma lipidomic differences between sepsis patients and healthy controls. The PCA results indicated clear separation between groups (P = 3.1e−16) (**Figure 3a**). A total of 603 differentially expressed lipid molecules (|FC| > 1.3, P < 0.05) were identified between the sepsis and healthy control groups, with 35% of lipids reduced, 12% increased, and 53% unchanged (**Figure 3b**). Detailed lipid species distributions within each major category (for categories with ≥10 species) revealed that, PG and LacCer were elevated during sepsis, whereas PC, CE, and LPC were notably reduced. (**Figure 3c**).

We further analyzed the correlation between 38 lipid subclasses and age in both healthy controls and sepsis patients. Our findings revealed that the majority of lipid subclasses exhibited varying correlations with age across different health states, with some even showing opposite trends. HexCer correlated positively with age in HC and negatively with age in Sepsis; LPC correlated negatively with age in HC and positively correlated with age in Sepsis. This suggests that the pathological state of sepsis differentially impacts lipid metabolism in patients of different ages(**Figure 3d**).

All blood samples were collected at hospital admission prior to the initiation of systematic treatment—including antibiotic therapy and other clinical interventions. This sampling approach minimizes acute in-hospital treatment effects on lipidomic profiles; however, potential influences from pre-existing comorbidities and pre-admission medications cannot be entirely excluded.

To evaluate the potential confounding effect of metabolic comorbidities, we conducted subgroup principal component analysis (PCA) comparing lipidomic profiles between patients with and without hypertension or diabetes. No significant separation was observed between these subgroups, either in the full lipidome (P = 0.939) or in the sepsis-specific lipid subset (P = 0.894) (**Supplementary Figure S1**), indicating that the lipid alterations identified were primarily attributable to sepsis itself rather than to underlying metabolic disorders.

Given the high prevalence of pulmonary infection among sepsis cases in our cohort, we further performed subgroup PCA to compare lipidomic profiles between patients with pulmonary versus non-pulmonary infection sources. The global lipidome (n = 1,277 lipids) showed no significant separation between groups (P = 0.762). Similarly, focused analysis of sepsis-altered lipids (n = 603) revealed overlapping distributions (P = 0.851) (**Supplementary Figure S2**). The absence of distinct clustering suggests that the site of infection does not substantially influence the core sepsis-associated lipidomic signature, supporting the systemic nature of lipid dysregulation in sepsis irrespective of the primary infection origin.

### Age stratification analysis in sepsis patients

**Figure 4:**
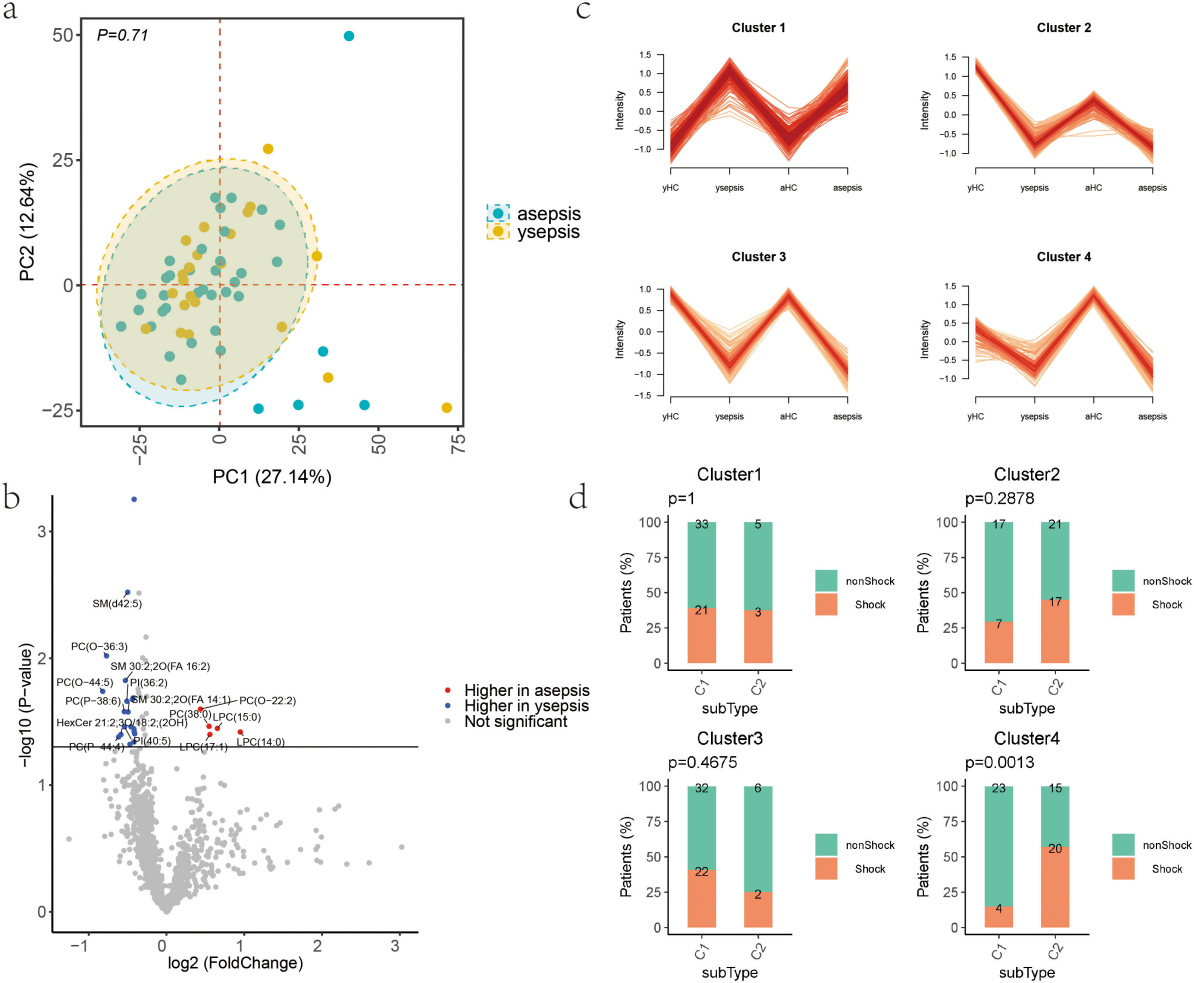
Lipid distribution differences between asepsis and ysepsis. **(a)** PCA plot of asepsis and ysepsis groups. Each point represents a sample, colored by group. Axes show PC1 and PC2. **(b)** Volcano plot of lipid abundance differences between asepsis and ysepsis. Points represent lipids, colored by significance. X-axis: fold change; Y-axis: -log10 P-value. Red: upregulated in asepsis; blue: downregulated. **(c)** Mfuzz clustering of 603 differential lipids (Sepsis_vs_HC) into 4 clusters. Trend plots show z-score normalized mean abundance per cluster. X-axis: sample groups; Y-axis: mean lipid abundance. **(d)** Bar plot showing shock patient counts in C1 and C2 subtypes across 4 clusters. Colors indicate shock status; X-axis: subtypes. P-values from Fisher’s test are shown.

We analyzed the age-related differences in sepsis patients by stratifying them into two groups: 41 cases of aging sepsis (asepsis) and 21 cases of young sepsis (ysepsis). No significant differences were observed between the asepsis and ysepsis groups in principal component analysis (PCA) (P > 0.05, **Figure 4a**). There were 24 differentially expressed lipid molecules between the asepsis and ysepsis groups (|FC| > 1.3, P < 0.05), with 5 upregulated and 19 downregulated in the asepsis compared to the ysepsis (**Figure 4b**).

To further elucidate the impact of age on plasma lipid profiles during the onset of sepsis, we conducted fuzzy clustering analysis based on the 603 differentially expressed lipid molecules identified between sepsis patients and healthy controls. Clustering trend plots were generated to visualize the distribution patterns of mean lipid levels within each clustering module (Cluster) (**Figure 4c**). The results showed that in Cluster1, both ysepsis and asepsis were upregulated compared to yHC and aHC. In Cluster2, Cluster3, and Cluster4, both ysepsis and asepsis were downregulated compared to yHC and aHC, but there were differences among the clusters. Specifically, in Cluster2, the lipid abundance in yHC was higher than that in aHC, and the changes in ysepsis were greater than those in asepsis. In Cluster3, the changes were similar in magnitude, while in Cluster4, the changes in ysepsis were smaller than those in asepsis.

Using the lipid molecules in each cluster, we performed consensus clustering on sepsis samples using the ConsensusClusterPlus package based on the Euclidean distance between samples, dividing the samples into two subtypes, C1 and C2. We counted the number of samples with shock and non-shock in each subtype and performed Fisher’s statistical test(**Figure 4d**). The subtyping based on the lipid molecules in Cluster4 showed a significant difference between shock and non - shock samples (P = 0.0013), with a higher proportion of shock patients in subtype C2 (C1:14.8%, C2:57.1%).

To elucidate the potential mechanisms underlying the lipid-immune axis, we performed KEGG pathway enrichment analysis based on DIA-based untargeted proteomic data from the C1 and C2 patient subgroups. The results revealed significant alterations in several key pathways: the ​​”Complement and coagulation cascades”​​ pathway and the ​​”Cholesterol metabolism”​​ pathway were both markedly dysregulated, suggesting a direct mechanistic link between lipid metabolic disturbance and age-related immune dysfunction. Furthermore, pronounced enrichment of the ​​”Chemical carcinogenesis - reactive oxygen species”​​ pathway indicated that oxidative stress may serve as a critical biological mediator connecting lipidomic alterations to immunosenescence(**Supplementary Figure S3**).

### Age-related Cluster4 and the severity of sepsis

**Figure 5:**
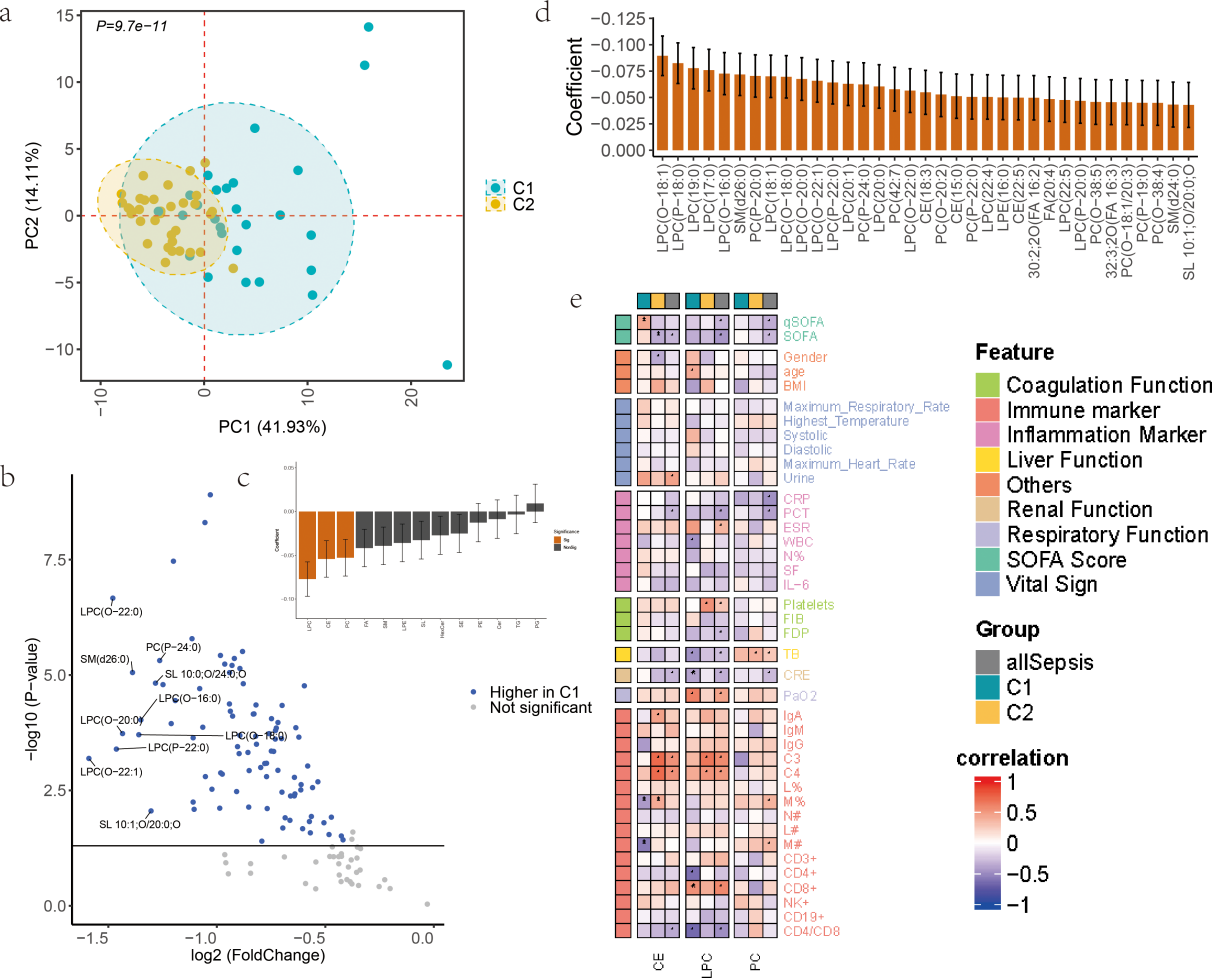
Lipid distribution differences between C1 and C2 subtypes in Cluster 4. **(a)** PCA plot of C1 and C2 subtypes. Each point represents a sample, colored by group. Axes show PC1 and PC2. P = 9.7e-11. **(b)** Volcano plot of lipid abundance differences between C1 and C2. Points represent lipids, colored by significance. X-axis: fold change; Y-axis: -log10 P-value. Blue: downregulated in C2. **(c, d)** Bar plots showing regression coefficients of lipid subclasses or molecules with SOFA scores. X-axis: lipid subclasses/molecules sorted by regression coefficient; Y-axis: regression coefficients. Error bars: ± SD. Orange highlights indicate P<0.05. **(e)** Heatmap of Pearson correlation coefficients between clinical indicators (rows) and lipid types (columns). * indicates P<0.05.

The median age in the C1 group was 67 (46-77), of which asepsis accounted for 55.6% (15/27); in the C2 group, the median age was 71 (54-80), of which asepsis accounted for 71.4% (25/35). Notably, gender distribution was comparable between C1 (66.7% male) and C2 (65.7% male), indicating minor sex-based bias in the lipid-defined subtypes. The clustering of the two subtypes (C1 and C2) in Cluster 4 exhibits significant differences, as demonstrated by the PCA plot (P = 9.7e-11) (**Figure 5a**). There are 92 differentially expressed lipid molecules (|FC| > 1.3, P < 0.05) between the C1 and C2 groups, all of which are downregulated in C2(**Figure 5b**). Based on these 92 differentially expressed lipid molecules, linear regression analysis was performed to examine their relationship with SOFA scores, both at the subclass and individual molecule levels. Using standardized lipid subclass or individual molecule expression as the independent variable and SOFA score as the dependent variable, a model was constructed using the Ordinary Least Squares (OLS) method to calculate regression coefficients and their corresponding P-values. The results revealed that LPC, CE, and PC subclasses were significantly negatively correlated with SOFA scores (P < 0.05) (**Figure 5c**), suggesting that increased expression of these subclasses may be associated with reduced SOFA scores and could play a positive role in improving patient prognosis. At the individual lipid molecule level, 35 lipid molecules showed significant negative correlations with SOFA scores (P < 0.05) (**Figure 5d**), further indicating their potential role in clinical outcomes.

We propose that C2 represents a more severe subtype of sepsis. Further investigation into the associations of LPC, CE, and PC with clinical indicators revealed differences between C1 and C2. Notably, CE showed a negative correlation with monocytes percentage in C1 but a positive correlation in C2. Both CE and LPC exhibited significant positive correlations with complement C3 and C4 in C2 group(**Figure 5e**).

**Figure 6:**
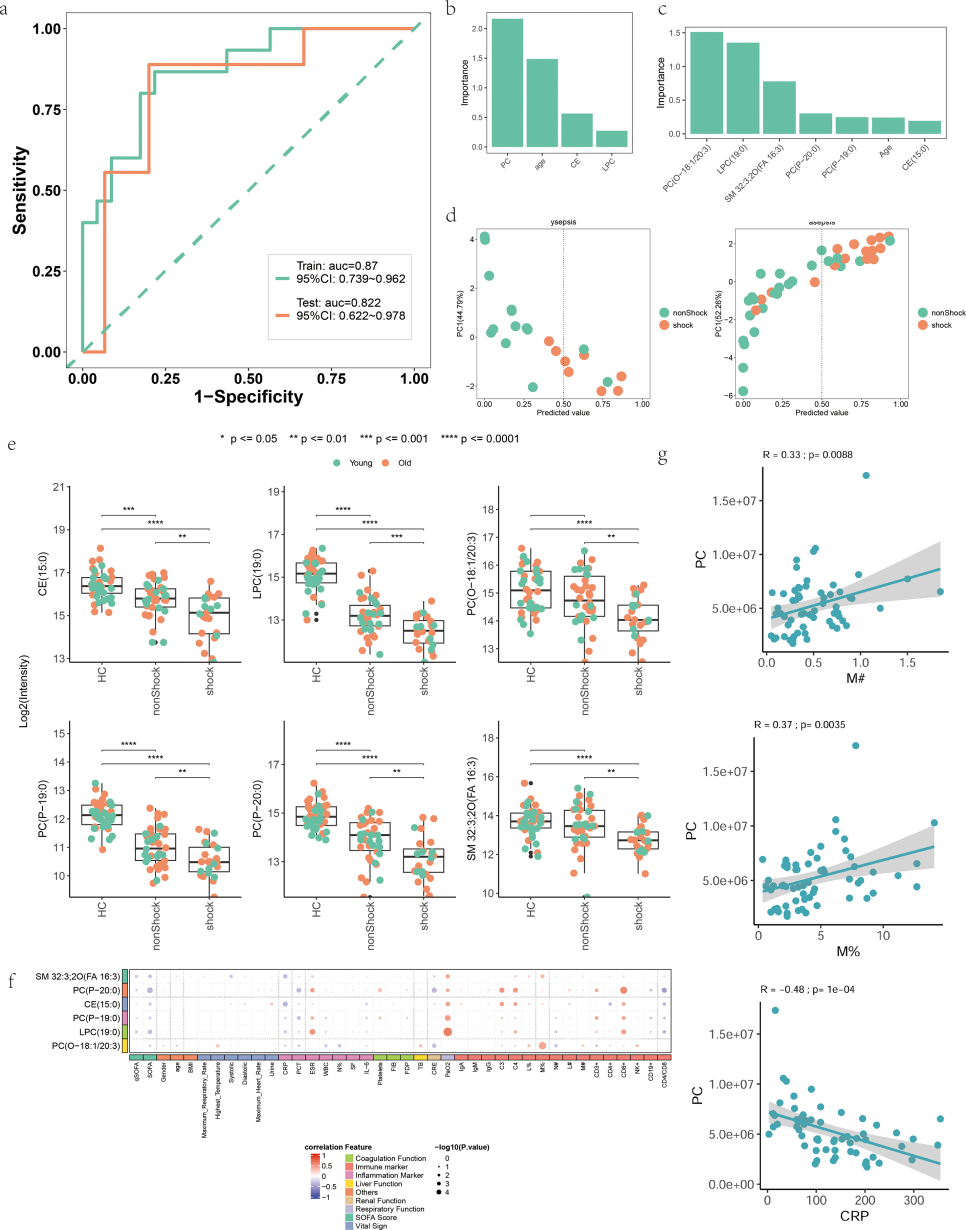
Lipid molecular model for predicting whether a patient will develop septic shock. **(a)** ROC curves for training and test sets. X-axis: 1-specificity (false positive); Y-axis: sensitivity (true positive). AUC indicates model performance. **(b)** Importance of lipid subclasses and age among 92 lipids related to SOFA scores. **(c)** Importance of final lipid molecules and age. X-axis: variables; Y-axis: importance (absolute t-statistic values). **(d)** PCA scatter plots of predicted vs. actual outcomes for aged and young samples. Y-axis: PC1; X-axis: predicted shock probability (threshold=0.5). **(e)** Bar plot of six lipid molecules in the model. X-axis: groups (HC, shock, non-shock), colored by age (young/old). Y-axis: log2-transformed lipid expression. P-values from t-tests are shown above. Black dots indicate observed values without jitter. **(f)** Bubble plot of correlations between six lipid molecules and clinical indicators. Bubble size: -log10(P-value); color: red (positive), blue (negative). **(g)** Correlation of PC lipid subclasses with clinical indicators. X-axis: clinical indicators; Y-axis: lipids. R: correlation coefficient; p: P-value.

We attempted to construct a classification model to predict whether sepsis patients would develop shock using the aforementioned 35 lipid molecules associated with SOFA scores, while also incorporating the age factor of the patients. We screened 35 molecules that showed significant linear correlations with SOFA and divided the samples into training and testing sets in a 6:4 ratio. Using the training set, we performed Lasso regression analysis on the 35 lipids and selected those lipids whose regression coefficients were not penalized to zero, ultimately identifying six lipid molecules: LPC(19:0), PC(P-19:0), SM 32:3;2O(FA 16:3), PC(P-20:0), PC(O-18:1/20:3), and CE(15:0) (**Figure 6c**). These six lipid molecules, along with age, were used to build the model. The final variables were used to establish a logistic regression model on the training set, employing a K-fold cross-validation method with k=5 and repeats=200. The absolute value of the t-statistic for each model parameter was used to represent the importance of the variables.

The results showed that the model could effectively distinguish between sepsis and septic shock patients, with an AUC of 0.87 for the training set and 0.822 for the testing set(**Figure 6a**). In the PCA plot, the horizontal coordinate represents the predicted probability of shock, with a threshold of 0.5 (values below 0.5 classified as nonShock and above 0.5 as shock). The actual distribution of shock patients largely aligned with the predictions, with most orange points located in the region above 0.5(**Figure 6d**). The model demonstrated predictive capability in both elderly and young sepsis patients. The ysepsis group accurately predicted 75%(6/8) of the shocked patients, and the asepsis group accurately predicted 81%(13/16) of the shocked patients.At the level of lipid subclasses to which the 35 molecules belong, PC contributed the most to the model, followed by age, CE, and LPC(**Figure 6b**). PC was positively correlated with the absolute monocyte count (R = 0.33, P = 0.0088) and monocyte percentage (R = 0.37, P = 0.0035) in sepsis patients, and negatively correlated with commonly used clinical inflammatory markers CRP (R=-0.48, P = 1e-04) and PCT (R=-0.36, P = 0.0056) (**Figure 6g**).

The distribution of the six lipid molecules across groups is shown in bar charts, and their correlations with clinical indicators are displayed in bubble plots. All six molecules had the highest plasma levels in healthy controls, reduced plasma levels in sepsis and the lowest plasma levels in septic shock(**Figure 6e**). They were negatively correlated with the inflammatory markers CRP, PCT and IL-6, positively correlated with complement C3, complement C4, percentage of lymphocytes, percentage of monocytes, and inversely correlated with absolute neutrophil count(**Figure 6f**).

### Utility of lipid model for predicting mortality in patients with sepsis

**Figure 7:**
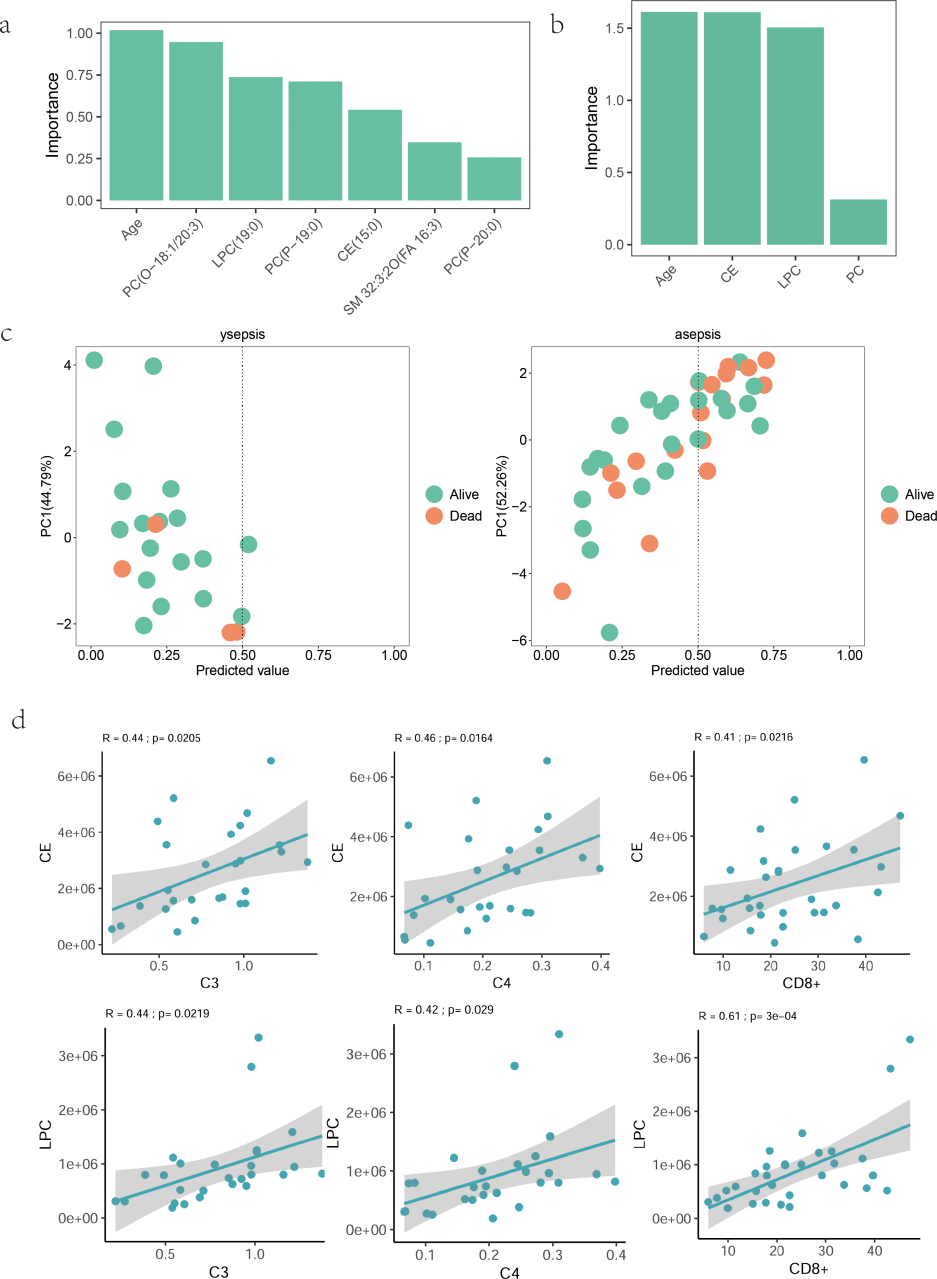
Utility of lipid model for predicting mortality. **(a, b)** Importance of final lipid molecules, subclasses, and age. X-axis: variables; Y-axis: importance (absolute t-statistic values). **(c)** PCA scatter plots of predicted vs. actual outcomes for aged and young samples. Y-axis: PC1; X-axis: predicted mortality probability (threshold=0.5). **(d)** Correlation of CE and LPC lipid subclasses with clinical indicators. X-axis: clinical indicators; Y-axis: lipids. R: correlation coefficient; p: P-value.

To further explore whether the above lipid model could identify patients’ in-hospital prognosis, we divided sepsis patients into in-hospital survival and in-hospital death groups to observe the classification efficacy of the model. We found that age was the most important predictor of patient prognosis, both at the lipid molecular level and at the lipid subclass level(**Figures 7a, b**). In young sepsis patients, the model was unable to predict patients’ in-hospital death, but in older sepsis patients, the model correctly identified 65% (11/17) of patients with in-hospital death(**Figure 7c**). We also analysed the association of the high-contributing lipid subclasses CE and LPC with clinical immune markers in patients, and both CE and LPC were positively correlated with complement C3, C4 and CD8+ cells, which suggests that complement C3, C4 and CD8+ cells correlate with the prognosis of elderly sepsis patients(**Figure 7d**).

## Discussion

This study provides the first systematic evidence of ​age-specific differences​ in plasma lipidomic profiles among sepsis patients. While no significant differences in overall lipid abundance were observed between elderly and young healthy controls, ​sepsis-induced lipidomic remodeling exhibited marked heterogeneity across age groups.Notably, we identified ​severity-associated lipid subclasses​ (PCs, CEs, and LPCs) with distinct age-associated patterns, suggesting that ​lipid metabolism and immune regulation may be differentially dysregulated in sepsis depending on patient age.

**Figure 8:**
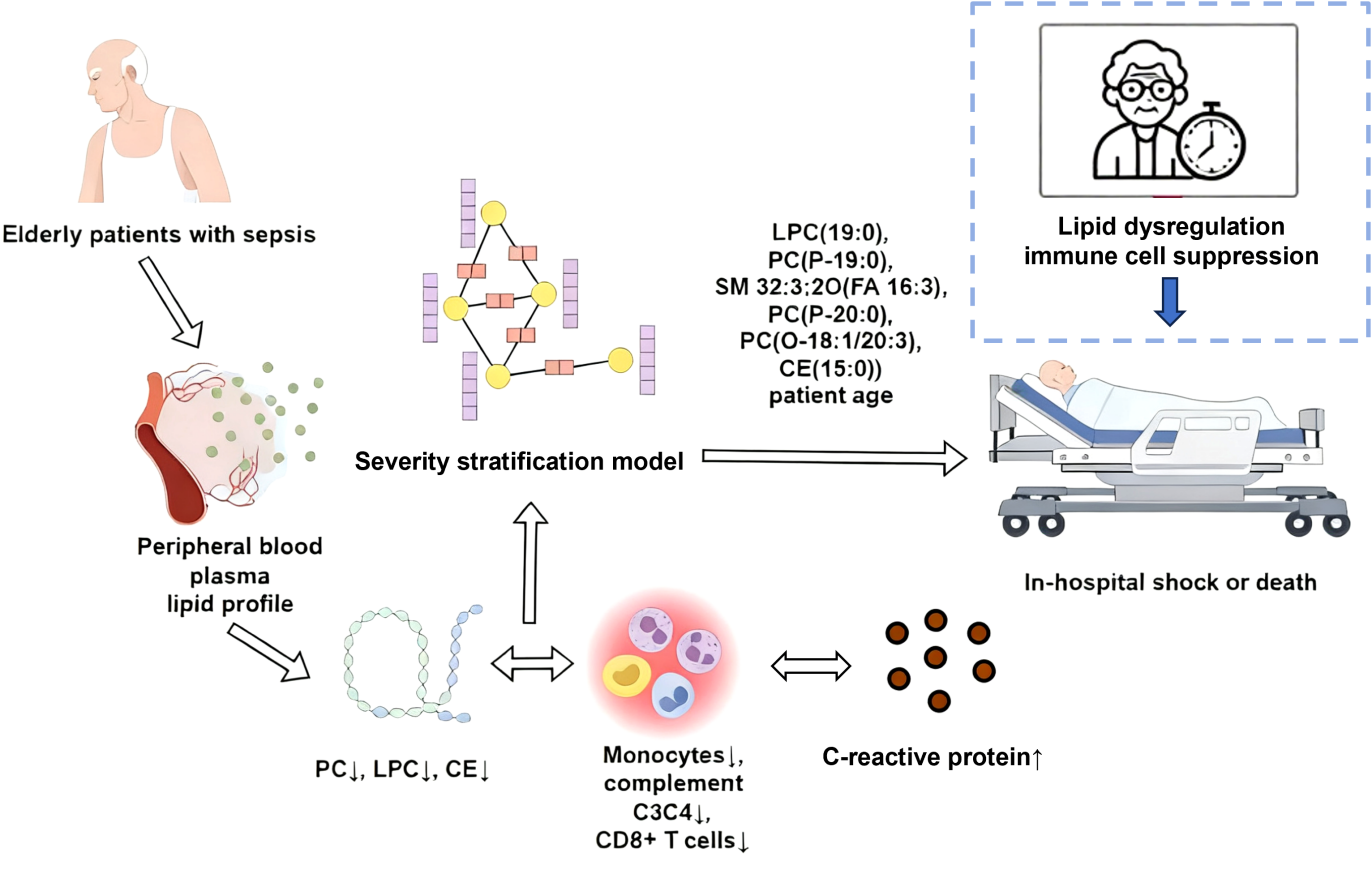
Schematic of the lipid-immune-clinical severity axis in elderly sepsis patients. Age-related immunosenescence establishes a baseline of vulnerability. During sepsis, this is exacerbated by a specific depletion of protective lipids (PC, LPC, CE), which in turn suppresses the function and abundance of key immune cells (monocytes, CD8^+^T cells, complement). This cascade of lipid dysregulation and immune impairment drives the progression to septic shock and increased mortality, illustrating the critical modifying role of immunosenescence in this patient population.

Our lipidomic analysis revealed that sepsis patients exhibit a marked reduction in key lipid subclasses—including phosphatidylcholines (PCs), cholesteryl esters (CEs), and lysophosphatidylcholines (LPCs)—which play critical roles in maintaining membrane stability, facilitating cholesterol transport, and modulating immune responses (23). Notably, clustering analysis of age-stratified lipid profiles identified Cluster 4 lipids as robust discriminators of sepsis subtypes (C1 vs. C2), with the C2 subtype demonstrating significantly higher shock incidence (57.1% vs. 14.8%). Building upon these findings, we developed a risk stratification model incorporating six lipid species [LPC(19:0), PC(P-19:0), SM 32:3;2O(FA 16:3), PC(P-20:0), PC(O-18:1/20:3), CE(15:0)] and patient age, which effectively stratified shock risk across age groups (AUC: 0.87 training, 0.82 validation) and predicted in-hospital mortality in elderly patients. Mechanistically, we observed that PC levels positively correlated with monocyte abundance, while CE and LPC levels were associated with complement factors (C3, C4) and CD8+ T cell proportions. These results collectively suggest that age-specific lipid remodeling may reflect distinct immunoregulatory pathways in sepsis, positioning lipidomic signatures as both biologically informative markers and clinically actionable predictors of outcomes (**Figure 8**).

These lipidomic changes may reflect broad disturbances in the metabolic and physiological states of sepsis patients (24, 25). Although it is challenging to directly attribute these changes to specific biological processes, we hypothesize that the reductions in PC, CE, and LPC are directly linked to the inflammatory responses associated with sepsis and the loss of cellular membrane integrity (26, 27). The importance of PC and LPC in cellular membranes and their roles in signal transduction suggest that their decreased levels may lead to cellular dysfunction, exacerbating the pathological state of sepsis (28, 29). Previous studies have indicated that LPC is essential for maintaining T-cell homeostasis (30), and its depletion leads to a reduction in the memory T-cell pool, thereby diminishing the secondary response to reinfections (23). Additionally, LPC induces chemotaxis and intracellular calcium influx in natural killer cells (31). In fact, LPC levels are inversely correlated with mortality in sepsis patients, indicating that the lower LPC levels observed in older sepsis patients in our study may predict poorer outcomes (32, 33).

Our analysis revealed a significant association between ether-linked (lyso)phosphatidylcholine species and SOFA scores, suggesting their potential role as biomarkers of sepsis severity. Ether phospholipids, particularly plasmalogens, are known to function as endogenous antioxidants that protect cellular membranes against oxidative stress—a hallmark of sepsis pathophysiology (34). Their depletion may reflect both increased consumption during radical scavenging and impaired synthesis due to peroxisomal dysfunction in severe sepsis (35). The more pronounced reduction of these ether lipids in elderly patients may further indicate an age-associated decline in antioxidant capacity, potentially contributing to the worse outcomes observed in this population (16, 36). While our study demonstrates strong clinical correlations, further mechanistic studies are needed to elucidate whether ether lipid supplementation could serve as a therapeutic strategy to mitigate oxidative damage in sepsis.

Moreover, a reduction in the CE may impair cholesterol transport and utilization, further weakening the immune response (37). CE is the storage form of cholesterol and is involved in cell membrane stability and cholesterol transport. CETP is responsible for the transport of cholesteryl esters in the body, and sepsis can lead to alterations in CETP expression (38), subsequently affecting cholesterol transport and CE synthesis (37, 39, 40). Venancio et al. proposed a potential protective role for CETP in regulating inflammatory responses, whereas Dusuel et al. suggested that human CETP may worsen outcomes in sepsis (41). Therefore, further studies are needed to elucidate the role of CE in sepsis and its relationship with CETP. Notably, the accumulation of CEs is crucial for sustaining T-cell proliferation in humans (42), and its decline may directly affect host immune efficacy.

Additionally, we investigated the relationship between lipid changes and immune response. CRP and PCT are widely used inflammatory markers in sepsis, whose levels are typically significantly elevated during infection and inflammatory responses (43, 44). The negative correlation between PC and CRP/PCT may be attributed to the anti-inflammatory effects of PC in inflammatory responses (45). Specifically, PC modulates membrane fluidity, thereby reducing the release of inflammatory mediators (46). Additionally, PC metabolites, such as LPC, can inhibit the activation of inflammatory cells, alleviating inflammatory responses (47). As a metabolite of PC, LPC exhibits multiple biological activities, including the regulation of inflammatory responses and cellular signaling. Both CE and LPC may influence the activation of the complement system by modulating the lipid microenvironment of cell membranes. For instance, LPC can induce the chemotaxis of neutrophils and monocytes, thereby promoting complement system activation (48). Meanwhile, CE metabolites may indirectly affect complement system function by regulating cholesterol transport. Furthermore, LPC can activate CD8+ T cells through its receptors (e.g., G2A) (49), while CE metabolites may indirectly modulate CD8+ T cell function by influencing cholesterol transport (50).

In our study, patients in subtype C2 exhibited higher severity and a greater proportion of elderly individuals. The lipid subclasses CE, PC, and LPC, which are associated with SOFA scores, were found to be decreased in this subtype. Given the correlations between these lipids and immune cells, we hypothesize that the pathogenesis of severe sepsis in the elderly may be related to reductions in complement C3, complement C4, monocytes, and CD8+ T cells. In the elderly, studies have indicated the presence of complement changes and a low-grade inflammatory state, which is the same trend as in the elderly critically ill patients in our study (51). This also suggests the reason for the higher probability of shock in elderly septic patients.These age-related differences in immune response mechanisms suggest that clinical treatment strategies for sepsis may need to be tailored on the basis of the patient’s age to better activate and maintain immune function (15, 52–54). Although our study revealed correlations between lipidomic profiles and certain immune parameters, we acknowledge that the immune profiling was limited in scope. Key markers of immune dysfunction in sepsis, such as HLA-DR expression on monocytes, T cell activation/exhaustion markers (e.g., CD28, CD38), were not assessed. Future studies incorporating broader immunophenotyping and cytokine profiling would provide a more comprehensive understanding of lipid-immune interactions in sepsis.

Despite presenting new insights, our study has several limitations. The sample size was relatively small, and there was no multicenter validation. Future studies should involve larger-scale clinical observations and validations while linking lipidomics with detailed immunosenescence profiling (16, 55).Although we minimized acute treatment effects through admission-time sampling, our study cannot exclude potential influences from long-term medications (e.g., statins, antihypertensive drugs) used for comorbid conditions. The lack of systematic medication data represents a limitation, particularly given that: Metformin and other antidiabetic medications are known to alter circulating lipid profiles (56). β-blockers and ACE inhibitors may affect phospholipid metabolism (57). Statins directly modulate cholesterol biosynthesis pathways (58). Future longitudinal studies with detailed medication records are needed to fully disentangle sepsis-specific lipid changes from pre-existing metabolic influences. While our study identified significant lipid alterations associated with sepsis severity and outcomes, the extent to which these changes are specific to sepsis—rather than general critical illness or systemic inflammation—requires further investigation.

Our study captures lipidomic profiles at a single timepoint during acute sepsis, aiming to identify state biomarkers associated with disease severity rather than to establish causal relationships. Importantly, while the temporal origin of these lipid alterations (cause vs. consequence) remains undetermined, their strong association with clinical outcomes supports their utility as prognostic indicators. To move beyond correlation and establish biological mechanism, future work will focus on functional validation using experimental models such as LPS-stimulated PBMCs or relevant cell lines. Specifically, we will investigate whether supplementation of key depleted lipids (e.g., LPC(19:0), CE(15:0)) can modulate critical immune functions including cytokine production, phagocytosis, and T-cell activation, thereby providing direct evidence for their immunomodulatory roles in sepsis.

In summary, our research highlights the complex relationship between lipidomic changes and clinical indicators in sepsis patients, particularly as influenced by age (59). These findings increase our understanding of the pathophysiology of sepsis and provide potential directions for future treatment strategies, especially when age-related factors are considered (60). Further investigations should explore immune response mechanisms across different age groups to offer new perspectives for sepsis treatment (61).

## Conclusion

Our study utilized nontargeted LC-MS/MS lipidomics to reveal significant age-specific differences in plasma lipid profiles among sepsis patients. Furthermore, we found that lipid molecules associated with age during sepsis correlate with high SOFA scores, potentially elucidating the reasons for poorer outcomes in older patients. We developed a robust risk stratification model integrating six lipid biomarkers (LPC(19:0), PC(P-19:0), SM 32:3;2O(FA 16:3), PC(P-20:0), PC(O-18:1/20:3), CE(15:0)) and patient age, accurately forecasting the risk of septic shock across age groups and predicting in-hospital mortality specifically in elderly patients. In sepsis subtypes characterized by older age and greater severity, decreased plasma levels of lipid subclasses CE, PC, and LPC likely reflect impaired immune functions, such as reductions in complement factors (C3, C4), monocytes, and CD8+ T cells, which may contribute mechanistically to the rapid clinical deterioration observed in elderly patients. Our findings highlight the clinical significance of plasma lipidomic profiling, pointing toward novel therapeutic interventions aimed at modulating host lipid metabolism to improve outcomes in sepsis. The stratified analysis underscores the importance of precise clinical diagnosis and treatment, as differing pathophysiological changes may require tailored interventions. Additionally, this research provides a reference for identifying lipid biomarkers associated with immunosenescence in disease states.

## Data Availability

The raw data supporting the conclusions of this article will be made available by the authors, without undue reservation.

## Supplementary material

Supplementary Figure 1 Lipidomic profiles stratified by comorbidity status. Principal component analysis (PCA) of lipidomic profiles from sepsis patients with (yellow) and without (blue) metabolic complications (hypertension and/or diabetes). ​PCA using all detected lipid species (n=1277) shows no significant separation between groups (P = 0.939). PCA using only sepsis-associated differential lipids (n=603) similarly demonstrates no significant group separation (P = 0.894). Percentages indicate the proportion of variance explained by each principal component (PC1/PC2). The absence of significant separation suggests that metabolic comorbidities did not substantially alter the sepsis-associated lipidomic signatures identified in this study.

Supplementary Figure 2 Lipidomic profiles stratified by infection site.​​ Principal component analysis (PCA) of plasma lipidomes from sepsis patients with pulmonary infections (red squares) versus others infections (blue circles). Global lipidome analysis (n=1,277 lipids) revealed no significant separation between groups (P = 0.762). Focused analysis of sepsis-altered lipids (n=603) similarly showed overlapping distributions (P = 0.851). The lack of distinct clustering suggests that infection site does not substantially influence the core sepsis-associated lipidomic signature, supporting the systemic nature of lipid dysregulation in sepsis regardless of primary infection origin.

Supplementary Figure 3 KEGG pathway enrichment analysis of proteomic profiles between C1 and C2 sepsis subgroups.​​ Bubble plot showing significantly enriched pathways (Y-axis) based on untargeted proteomics data. Bubble size corresponds to the number of proteins mapped to each pathway (Count), and color intensity represents the Ratio (proportion of significant proteins in the pathway). The X-axis indicates the statistical significance (P-value).

## References

[1] Schwarz B Sharma L Roberts L Peng X Bermejo S Leighton I . Cutting edge: severe SARS-coV-2 infection in humans is defined by a shift in the serum lipidome, resulting in dysregulation of eicosanoid immune mediators. J Immunol. (2021) 206:329–34. doi: 10.4049/jimmunol.2001025, PMID: 33277388 PMC7962598

[2] Brunker LB Boncyk CS Rengel KF Hughes CG . Elderly patients and management in intensive care units (ICU): clinical challenges. Clin Interv Aging. (2023) 18:93–112. doi: 10.2147/CIA.S365968, PMID: 36714685 PMC9879046

[3] Thavamani A Umapathi KK Dhanpalreddy H Khatana J Chotikanatis K Allareddy V . Epidemiology, clinical and microbiologic profile and risk factors for inpatient mortality in pediatric severe sepsis in the United States from 2003 to 2014: A large population analysis. Pediatr Infect Dis J. (2020) 39:781–8. doi: 10.1097/INF.0000000000002669, PMID: 32221163

[4] Hornburg D Wu S Moqri M Zhou X Contrepois K Bararpour N . Dynamic lipidome alterations associated with human health, disease and ageing. Nat Metab. (2023) 5:1578–94. doi: 10.1038/s42255-023-00880-1, PMID: 37697054 PMC10513930

[5] Li X Yin Z Yan W Wang M Chang C Guo C . Association between changes in plasma metabolism and clinical outcomes of sepsis. Emerg Med Int. (2023) 2023:2590115. doi: 10.1155/2023/2590115, PMID: 37346225 PMC10281824

[6] Lee EH Shin MH Park JM Lee SG Ku NS Kim YS . Diagnosis and mortality prediction of sepsis via lysophosphatidylcholine 16:0 measured by MALDI-TOF MS. Sci Rep. (2020) 10:13833. doi: 10.1038/s41598-020-70799-0, PMID: 32796893 PMC7427783

[7] Cambiaghi A Pinto BB Brunelli L Falcetta F Aletti F Bendjelid K . Characterization of a metabolomic profile associated with responsiveness to therapy in the acute phase of septic shock. Sci Rep. (2017) 7:9748. doi: 10.1038/s41598-017-09619-x, PMID: 28851978 PMC5575075

[8] Wei J Wong LC Boland S . Lipids as emerging biomarkers in neurodegenerative diseases. Int J Mol Sci. (2023) 25. doi: 10.3390/ijms25010131, PMID: 38203300 PMC10778656

[9] Santoro A Bientinesi E Monti D . Immunosenescence and inflammaging in the aging process: age-related diseases or longevity? Ageing Res Rev. (2021) 71:101422. doi: 10.1016/j.arr.2021.101422, PMID: 34391943

[10] Rogers AJ Leligdowicz A Contrepois K Jauregui A Vessel K Deiss TJ . Plasma metabolites in early sepsis identify distinct clusters defined by plasma lipids. Crit Care Explor. (2021) 3:e0478. doi: 10.1097/CCE.0000000000000478, PMID: 34345827 PMC8323800

[11] Arshad H Alfonso JCL Franke R Michaelis K Araujo L Habib A . Decreased plasma phospholipid concentrations and increased acid sphingomyelinase activity are accurate biomarkers for community-acquired pneumonia. J Transl Med. (2019) 17:365. doi: 10.1186/s12967-019-2112-z, PMID: 31711507 PMC6849224

[12] Meng H Sengupta A Ricciotti E Mrcela A Mathew D Mazaleuskaya LL . Deep phenotyping of the lipidomic response in COVID-19 and non-COVID-19 sepsis. Clin Transl Med. (2023) 13:e1440. doi: 10.1002/ctm2.1440, PMID: 37948331 PMC10637636

[13] Acosta-Ampudia Y Monsalve DM Rojas M Rodriguez Y Gallo JE Salazar-Uribe JC . COVID-19 convalescent plasma composition and immunological effects in severe patients. J Autoimmun. (2021) 118:102598. doi: 10.1016/j.jaut.2021.102598, PMID: 33524876 PMC7826092

[14] Ding W Xu S Zhou B Zhou R Liu P Hui X . Dynamic plasma lipidomic analysis revealed cholesterol ester and amides associated with sepsis development in critically ill patients after cardiovascular surgery with cardiopulmonary bypass. J Pers Med. (2022) 12. doi: 10.3390/jpm12111838, PMID: 36579569 PMC9693300

[15] Rowe TA Mckoy JM . Sepsis in older adults. Infect Dis Clin North Am. (2017) 31:731–42. doi: 10.1016/j.idc.2017.07.010, PMID: 29079157

[16] Rodrigues LP Teixeira VR Alencar-Silva T Simonassi-Paiva B Pereira RW Pogue R . Hallmarks of aging and immunosenescence: Connecting the dots. Cytokine Growth Factor Rev. (2021) 59:9–21. doi: 10.1016/j.cytogfr.2021.01.006, PMID: 33551332

[17] Yang WH Heithoff DM Aziz PV Haslund-Gourley B Westman JS Narisawa S . Accelerated aging and clearance of host anti-inflammatory enzymes by discrete pathogens fuels sepsis. Cell Host Microbe. (2018) 24:500–13 e5. doi: 10.1016/j.chom.2018.09.011, PMID: 30308156 PMC6223661

[18] Stephenson DJ Hoeferlin LA Chalfant CE . Lipidomics in translational research and the clinical significance of lipid-based biomarkers. Transl Res. (2017) 189:13–29. doi: 10.1016/j.trsl.2017.06.006, PMID: 28668521 PMC5659874

[19] Langley RJ Tsalik EL Van Velkinburgh JC Glickman SW Rice BJ Wang C . An integrated clinico-metabolomic model improves prediction of death in sepsis. Sci Transl Med. (2013) 5:195ra95. doi: 10.1126/scitranslmed.3005893, PMID: 23884467 PMC3924586

[20] Birner C Mester P Liebisch G Horing M Schmid S Muller M . Lipid metabolism disorders as diagnostic biosignatures in sepsis. Infect Dis Rep. (2024) 16:806–19. doi: 10.3390/idr16050062, PMID: 39311203 PMC11417812

[21] Sharma NK Ferreira BL Tashima AK Brunialti MKC Torquato RJS Bafi A . Lipid metabolism impairment in patients with sepsis secondary to hospital acquired pneumonia, a proteomic analysis. Clin Proteomics. (2019) 16:29. doi: 10.1186/s12014-019-9252-2, PMID: 31341447 PMC6631513

[22] Gonzalez-Covarrubias V . Lipidomics in longevity and healthy aging. Biogerontology. (2013) 14:663–72. doi: 10.1007/s10522-013-9450-7, PMID: 23948799

[23] Kabarowski JH . G2A and LPC: regulatory functions in immunity. Prostaglandins Other Lipid Mediat. (2009) 89:73–81. doi: 10.1016/j.prostaglandins.2009.04.007, PMID: 19383550 PMC2740801

[24] Chung HY Hupe DC Otto GP Sprenger M Bunck AC Dorer MJ . Acid sphingomyelinase promotes endothelial stress response in systemic inflammation and sepsis. Mol Med. (2016) 22:412–23. doi: 10.2119/molmed.2016.00140, PMID: 27341515 PMC5072414

[25] Sheikh AM Ochi H Masuda J . Lysophosphatidylcholine inhibits T cell-specific CXC chemokines IP-10, MIG, and I-TAC expression induced by IFN-gamma in human endothelial cells. Ann N Y Acad Sci. (2001) 947:306–7. doi: 10.1111/j.1749-6632.2001.tb03952.x, PMID: 11795279

[26] Liang S Zhou J Cao C Liu Y Ming S Liu X . GITR exacerbates lysophosphatidylcholine-induced macrophage pyroptosis in sepsis via posttranslational regulation of NLRP3. Cell Mol Immunol. (2024) 21:674–88. doi: 10.1038/s41423-024-01170-w, PMID: 38740925 PMC11214634

[27] Trinder M Boyd JH Brunham LR . Molecular regulation of plasma lipid levels during systemic inflammation and sepsis. Curr Opin Lipidol. (2019) 30:108–16. doi: 10.1097/MOL.0000000000000577, PMID: 30649022

[28] Ahn WG Jung JS Kwon HY Song DK . Alteration of lysophosphatidylcholine-related metabolic parameters in the plasma of mice with experimental sepsis. Inflammation. (2017) 40:537–45. doi: 10.1007/s10753-016-0500-6, PMID: 28028754

[29] Whalen MM Doshi RN Bader BW Bankhurst AD . Lysophosphatidylcholine and arachidonic acid are required in the cytotoxic response of human natural killer cells to tumor target cells. Cell Physiol Biochem. (1999) 9:297–309. doi: 10.1159/000016324, PMID: 10749996

[30] Piccirillo AR Hyzny EJ Beppu LY Menk AV Wallace CT Hawse WF . The lysophosphatidylcholine transporter MFSD2A is essential for CD8(+) memory T cell maintenance and secondary response to infection. J Immunol. (2019) 203:117–26. doi: 10.4049/jimmunol.1801585, PMID: 31127034 PMC6581627

[31] Jin Y Damaj BB Maghazachi AA . Human resting CD16-, CD16+ and IL-2-, IL-12-, IL-15- or IFN-alpha-activated natural killer cells differentially respond to sphingosylphosphorylcholine, lysophosphatidylcholine and platelet-activating factor. Eur J Immunol. (2005) 35:2699–708. doi: 10.1002/eji.200526129, PMID: 16078278

[32] Hara Y Kusumi Y Mitsumata M Li XK Fujino M . Lysophosphatidylcholine upregulates LOX-1, chemokine receptors, and activation-related transcription factors in human T-cell line Jurkat. J Thromb Thrombolysis. (2008) 26:113–8. doi: 10.1007/s11239-007-0158-x, PMID: 17963022

[33] Trovato FM Zia R Artru F Mujib S Jerome E Cavazza A . Lysophosphatidylcholines modulate immunoregulatory checkpoints in peripheral monocytes and are associated with mortality in people with acute liver failure. J Hepatol. (2023) 78:558–73. doi: 10.1016/j.jhep.2022.10.031, PMID: 36370949

[34] Henning T Kochlik B Kusch P Strauss M Juric V Pignitter M . Pre-operative assessment of micronutrients, amino acids, phospholipids and oxidative stress in bariatric surgery candidates. Antioxidants (Basel). (2022) 11. doi: 10.3390/antiox11040774, PMID: 35453460 PMC9031169

[35] Zuo WY Wen M Zhao YC Li XY Xue CH Yanagita T . Effects of short-term supplementation with DHA-enriched phosphatidylcholine and phosphatidylserine on lipid profiles in the brain and liver of n-3 PUFA-deficient mice in early life after weaning. J Sci Food Agric. (2024) 104:7939–52. doi: 10.1002/jsfa.13625, PMID: 38843481

[36] Rajakumari S Srivastava S . Aging and beta3-adrenergic stimulation alter mitochondrial lipidome of adipose tissue. Biochim Biophys Acta Mol Cell Biol Lipids. (2021) 1866:158922. doi: 10.1016/j.bbalip.2021.158922, PMID: 33713833

[37] Dusuel A Deckert V Pais De Barros JP Van Dongen K Choubley H Charron E . Human cholesteryl ester transfer protein lacks lipopolysaccharide transfer activity, but worsens inflammation and sepsis outcomes in mice. J Lipid Res. (2021) 62:100011. doi: 10.1194/jlr.RA120000704, PMID: 33500240 PMC7859855

[38] Perez-Hernandez EG De La Puente-Diaz De Leon V Luna-Reyes I Delgado-Coello B Sifuentes-Osornio J Mas-Oliva J . The cholesteryl-ester transfer protein isoform (CETPI) and derived peptides: new targets in the study of Gram-negative sepsis. Mol Med. (2022) 28:157. doi: 10.1186/s10020-022-00585-3, PMID: 36536294 PMC9764724

[39] Deng H Liang WY Chen LQ Yuen TH Sahin B Vasilescu DM . CETP inhibition enhances monocyte activation and bacterial clearance and reduces streptococcus pneumonia-associated mortality in mice. JCI Insight. (2024) 9. doi: 10.1172/jci.insight.173205, PMID: 38646937 PMC11141867

[40] Trinder M Genga KR Kong HJ Blauw LL Lo C Li X . Cholesteryl ester transfer protein influences high-density lipoprotein levels and survival in sepsis. Am J Respir Crit Care Med. (2019) 199:854–62. doi: 10.1164/rccm.201806-1157OC, PMID: 30321485

[41] Venancio TM Machado RM Castoldi A Amano MT Nunes VS Quintao EC . CETP lowers TLR4 expression which attenuates the inflammatory response induced by LPS and polymicrobial sepsis. Mediators Inflammation. (2016) 2016:1784014. doi: 10.1155/2016/1784014, PMID: 27293313 PMC4880711

[42] Hofmaenner DA Kleyman A Press A Bauer M Singer M . The many roles of cholesterol in sepsis: A review. Am J Respir Crit Care Med. (2022) 205:388–96. doi: 10.1164/rccm.202105-1197TR, PMID: 34715007 PMC8886946

[43] Tang Y Fung E Xu A Lan HY . C-reactive protein and ageing. Clin Exp Pharmacol Physiol. (2017) 44 Suppl 1:9–14. doi: 10.1111/1440-1681.12758, PMID: 28378496

[44] Stocker M Van Herk W El Helou S Dutta S Schuerman F Van Den Tooren-de Groot RK . C-reactive protein, procalcitonin, and white blood count to rule out neonatal early-onset sepsis within 36 hours: A secondary analysis of the neonatal procalcitonin intervention study. Clin Infect Dis. (2021) 73:e383–e90. doi: 10.1093/cid/ciaa876, PMID: 32881994

[45] Ai R Xu J Ji G Cui B . Exploring the phosphatidylcholine in inflammatory bowel disease: potential mechanisms and therapeutic interventions. Curr Pharm Des. (2022) 28:3486–91. doi: 10.2174/1381612829666221124112803, PMID: 36424797

[46] Papangelis A Ulven T . Synthesis of lysophosphatidylcholine and mixed phosphatidylcholine. J Org Chem. (2022) 87:8194–7. doi: 10.1021/acs.joc.2c00335, PMID: 35649118

[47] Law SH Chan ML Marathe GK Parveen F Chen CH Ke LY . An updated review of lysophosphatidylcholine metabolism in human diseases. Int J Mol Sci. (2019) 20. doi: 10.3390/ijms20051149, PMID: 30845751 PMC6429061

[48] Matsumoto T Kobayashi T Kamata K . Role of lysophosphatidylcholine (LPC) in atherosclerosis. Curr Med Chem. (2007) 14:3209–20. doi: 10.2174/092986707782793899, PMID: 18220755

[49] Kabarowski JH Zhu K Le LQ Witte ON Xu Y . Lysophosphatidylcholine as a ligand for the immunoregulatory receptor G2A. Science. (2001) 293:702–5. doi: 10.1126/science.1061781, PMID: 11474113

[50] Su L Huang Y Zhu Y Xia L Wang R Xiao K . Discrimination of sepsis stage metabolic profiles with an LC/MS-MS-based metabolomics approach. BMJ Open Respir Res. (2014) 1:e000056. doi: 10.1136/bmjresp-2014-000056, PMID: 25553245 PMC4265126

[51] Sun Y Lu J Wu J Qi X Huang Y Lin K . Potential mechanism of CARD16 protein action and susceptibility to sepsis in the elderly infected population: Through transcriptome analysis of blood. Int J Biol Macromol. (2024) 281:136578. doi: 10.1016/j.ijbiomac.2024.136578, PMID: 39406325

[52] Chang DH Deng H Matthews P Krasovsky J Ragupathi G Spisek R . Inflammation-associated lysophospholipids as ligands for CD1d-restricted T cells in human cancer. Blood. (2008) 112:1308–16. doi: 10.1182/blood-2008-04-149831, PMID: 18535199 PMC2515141

[53] Trinder M Wang Y Madsen CM Ponomarev T Bohunek L Daisely BA . Inhibition of cholesteryl ester transfer protein preserves high-density lipoprotein cholesterol and improves survival in sepsis. Circulation. (2021) 143:921–34. doi: 10.1161/CIRCULATIONAHA.120.048568, PMID: 33228395

[54] Fox LM Cox DG Lockridge JL Wang X Chen X Scharf L . Recognition of lyso-phospholipids by human natural killer T lymphocytes. PloS Biol. (2009) 7:e1000228. doi: 10.1371/journal.pbio.1000228, PMID: 19859526 PMC2760207

[55] Han X Gross RW . The foundations and development of lipidomics. J Lipid Res. (2022) 63:100164. doi: 10.1016/j.jlr.2021.100164, PMID: 34953866 PMC8953652

[56] Pradas I Rovira-Llopis S Naudi A Banuls C Rocha M Hernandez-Mijares A . Metformin induces lipid changes on sphingolipid species and oxidized lipids in polycystic ovary syndrome women. Sci Rep. (2019) 9:16033. doi: 10.1038/s41598-019-52263-w, PMID: 31690730 PMC6831788

[57] Hayase N Satomi M Hara A Awaya T Shimizu K Matsubara K . Protective effects of quinaprilat and trandolaprilat, active metabolites of quinapril and trandolapril, on hemolysis induced by lysophosphatidylcholine in human erythrocytes. Biol Pharm Bull. (2003) 26:712–6. doi: 10.1248/bpb.26.712, PMID: 12736518

[58] Siniscalchi C Basaglia M Riva M Meschi M Meschi T Castaldo G . Statins effects on blood clotting: A review. Cells. (2023) 12. doi: 10.3390/cells12232719, PMID: 38067146 PMC10706238

[59] Lopez-Sagaseta J Sibener LV Kung JE Gumperz J Adams EJ . Lysophospholipid presentation by CD1d and recognition by a human Natural Killer T-cell receptor. EMBO J. (2012) 31:2047–59. doi: 10.1038/emboj.2012.54, PMID: 22395072 PMC3343337

[60] Liu Z Liang Q Ren Y Guo C Ge X Wang L . Immunosenescence: molecular mechanisms and diseases. Signal Transduct Target Ther. (2023) 8:200. doi: 10.1038/s41392-023-01451-2, PMID: 37179335 PMC10182360

[61] Mecatti GC Sanchez-Vinces S Fernandes A Messias MCF De Santis GKD Porcari AM . Potential lipid signatures for diagnosis and prognosis of sepsis and systemic inflammatory response syndrome. Metabolites. (2020) 10. doi: 10.3390/metabo10090359, PMID: 32882869 PMC7570015

