# Supplementary figures and images for: Plasma lipidomic signatures reveal age-associated patterns of septic shock risk and immune dysregulation in sepsis
Source: Front Immunol. 2025 Nov 19;16:1659425. doi: 10.3389/fimmu.2025.1659425 (PMC12672909; doi:10.3389/fimmu.2025.1659425)

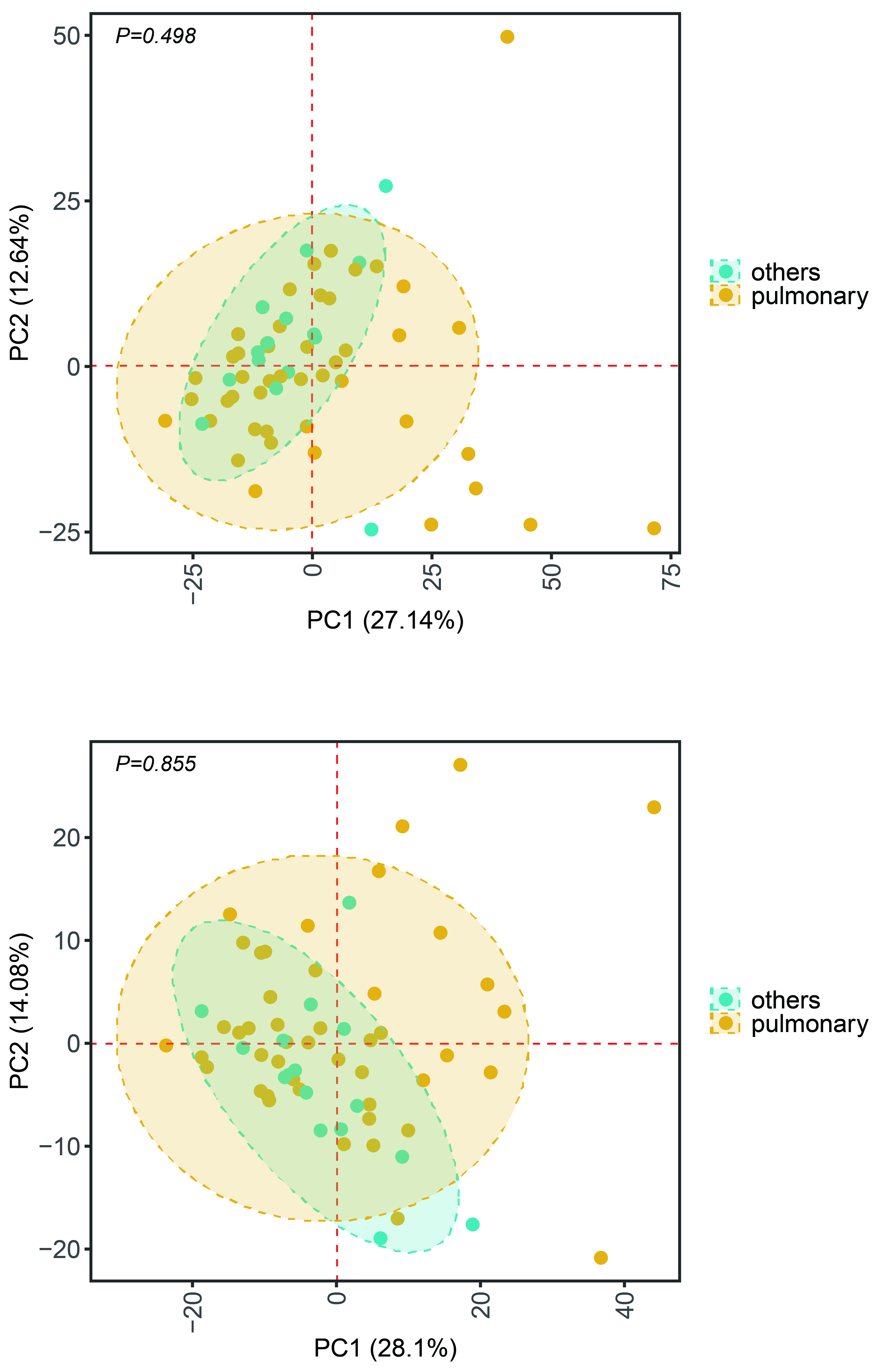

Supplement: Supplementary Figure 1 — Lipidomic profiles stratified by comorbidity status. Principal component analysis (PCA) of lipidomic profiles from sepsis patients with (yellow) and without (blue) metabolic complications (hypertension and/or diabetes). ​PCA using all detected lipid species (n=1277) shows no significant separation between groups (P = 0.939). PCA using only sepsis-associated differential lipids (n=603) similarly demonstrates no significant group separation (P = 0.894). Percentages indicate the proportion of variance explained by each principal component (PC1/PC2). The absence of significant separation suggests that metabolic comorbidities did not substantially alter the sepsis-associated lipidomic signatures identified in this study. [file Image1.tif]

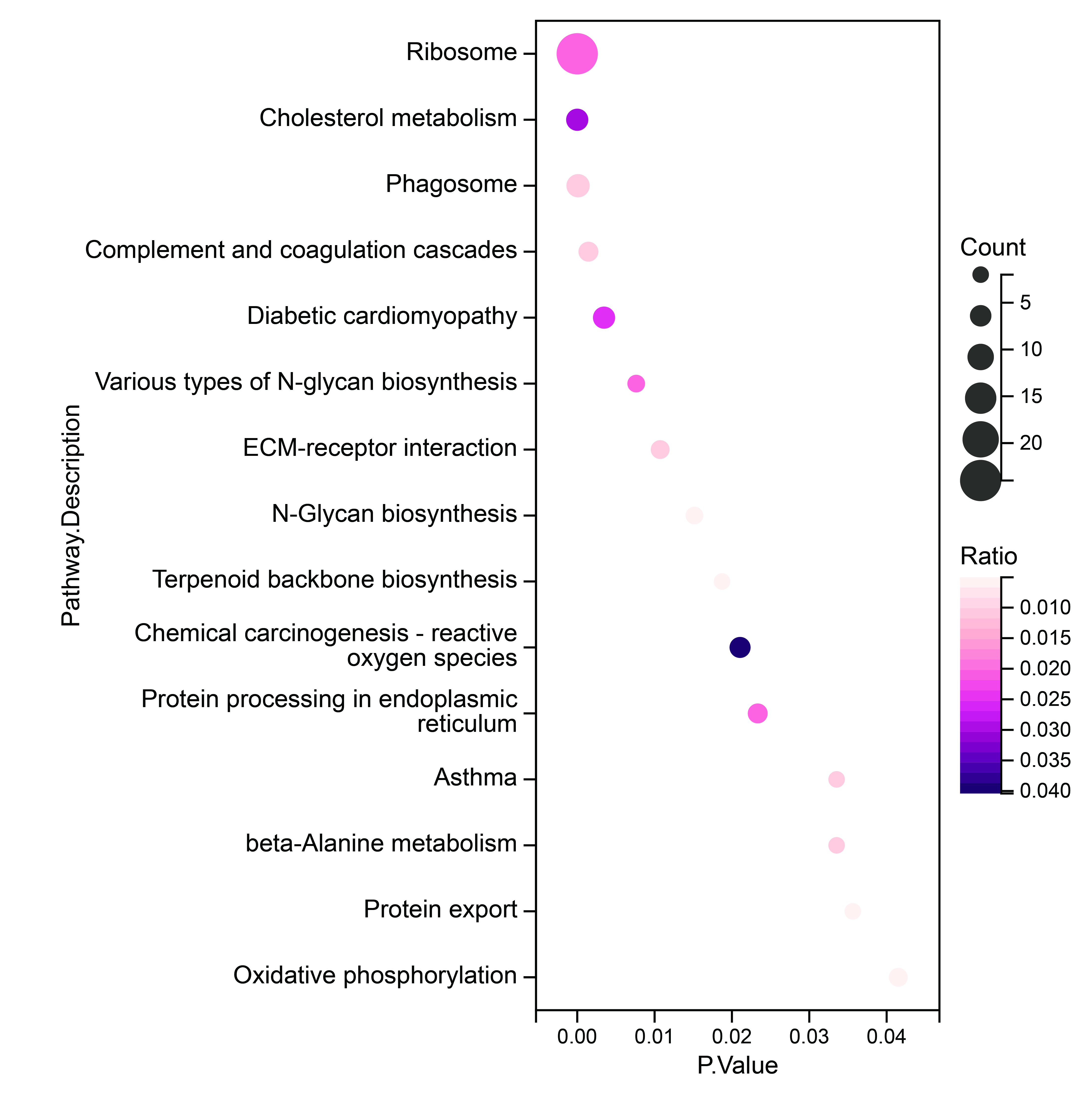

Supplement: Supplementary Figure 3 — KEGG pathway enrichment analysis of proteomic profiles between C1 and C2 sepsis subgroups.​​ Bubble plot showing significantly enriched pathways (Y-axis) based on untargeted proteomics data. Bubble size corresponds to the number of proteins mapped to each pathway (Count), and color intensity represents the Ratio (proportion of significant proteins in the pathway). The X-axis indicates the statistical significance (P-value). [file Image3.tif]
